# Enhancing Student Engagement Through Active Teaching-Learning Approaches Among First-Year Medical Undergraduates

**DOI:** 10.7759/cureus.85921

**Published:** 2025-06-13

**Authors:** Harish Rangareddy, K S Govinda Swamy, Ashakiran S, Mahalaxmi S Petimani

**Affiliations:** 1 Biochemistry, Haveri Institute of Medical Sciences, Haveri, IND; 2 Biochemistry, Shimoga Institute of Medical Sciences, Shimoga, IND

**Keywords:** active learning methods, critical thinking, medical student engagement, problem-based learning (pbl), self-directed learning

## Abstract

Background

Active teaching-learning approaches involve students in the learning process, promoting critical thinking, problem-solving, and knowledge retention. These methods contrast with traditional didactic lectures, which focus on passive listening. This study aims to evaluate the perceptions and effectiveness of active teaching-learning methods among first-year medical students.

Methodology

A cross-sectional survey of 113 first-year medical students was conducted using a validated questionnaire (Cronbach’s alpha = 0.873). Active teaching-learning methods, viz., small group discussions, case-based learning, flipped classroom, and early clinical exposure sessions, were implemented to enhance engagement among first-year MBBS students. The survey collected data on engagement, retention, and critical thinking through Likert-scale questions and open-ended feedback. Data were analyzed using SPSS version 16 (SPSS Inc., Chicago, IL, USA).

Results

Overall, 79.7% of students agreed that active teaching methods enhanced engagement, while 75.2% reported improved retention. Further, 83.2% felt their clinical reasoning skills benefited. Students emphasized the importance of interactive sessions and real-life clinical case discussions.

Conclusions

Active teaching-learning approaches improve engagement and critical thinking, aligning with the needs of modern medical education. Integration of these methods into the curriculum, alongside adequate resource allocation, is crucial for long-term success.

## Introduction

Medical education has evolved significantly, with an increasing emphasis on active learning strategies to meet the dynamic demands of healthcare systems. Active teaching-learning methods involve students in meaningful learning activities that go beyond passive listening, fostering higher-order thinking skills such as analysis, synthesis, and evaluation [1,2]. In the traditional classroom, students often rely on rote memorization with minimal opportunities for interaction or practical application. This approach, while foundational, may limit the development of critical skills required for clinical practice [3]. Active learning, on the other hand, incorporates techniques such as group discussions, problem-solving, role-playing, and self-directed learning modules, enabling students to apply theoretical knowledge to practical scenarios [2].

This study aimed to explore the perceptions of first-year medical students regarding active teaching-learning methods and evaluate their impact on classroom engagement and learning outcomes. By understanding these perceptions, medical educators can better align teaching strategies with student needs, ultimately enhancing the quality of medical education.

This study was presented as an oral paper at the International Conference on Health Professions Education (ICHPE) 2024 held on December 13-14, 2024, at Sri Devaraj Urs Academy of Higher Education and Research, Kolar, India.

## Materials and methods

Study design

This cross-sectional study was conducted at Haveri Institute of Medical Sciences, Karnataka, India, among first-year medical students who had recently completed their first professional examinations during the period from September 2024 to November 2024.

Study population and sampling

The study targeted first-year medical students, with a calculated sample size of 96. Students were briefed in person by the researchers about the study goals and procedures. The sample size for the survey was calculated considering the finite population of first-year medical students in Karnataka using the following equations by Raosoft (R) software calculator (Raosoft Inc., Seattle, Washington) [4]: x  =  Z (c/100)2r (100-r) and n  =  Nx/((N-1) E2 + x), where N is the population size, E is the margin of error (10%), r is the fraction of responses (50%), and Z (c/100) is the critical value for the confidence level c (5%). The overall first-year medical undergraduate admission to various medical colleges in the state of Karnataka at the time of the study was 11,745, and 150 students’ annual admissions to Haveri Institute of Medical Sciences, Haveri, for the first year were filled. A margin of error of 10% was initially assumed, and the sample size obtained was 96.

Ethical clearance for the study was obtained from the Institutional Ethics Committee of Haveri Institute of Medical Sciences (approval number: HIMS/IEC/2024/02). In this cross-sectional descriptive study, it was decided to proceed with purposive sampling of first-year medical students who had recently completed their first professional examinations at the time of the study. Second-year students were excluded as the study was specifically designed to target first-year medical undergraduates because this cohort is at a critical transitional stage, from passive learning in high school to more self-directed, active learning in medical school. Moreover, including them would have introduced variability in the teaching content and assessment methods, potentially confounding the study outcomes.

Data collection tool

During the academic year 2023-2024, a variety of active teaching-learning methods were systematically implemented for the first-year MBBS students at our institution. These included small group discussions, case-based learning, self-directed learning, and the flipped classroom approach. Additionally, interactive strategies such as student-led seminars, quizzes, concept mapping, time management activity, and early clinical exposure were incorporated to promote deeper understanding and critical thinking. These sessions were aligned with the Competency-Based Medical Education curriculum prescribed by the National Medical Commission (NMC) of India and integrated into both theoretical and practical components across first-year subjects, i.e., anatomy, physiology, and biochemistry. As mandated by the NMC, faculty members undergo training in Basic Course Medical Education and were already sensitized to the active teaching-learning methods before implementation, and structured plans ensured consistency in delivery. The effectiveness of these approaches was subsequently assessed through student feedback collected via a validated questionnaire. Data were collected using a self-administered, faculty-validated questionnaire. The questionnaire was designed to assess students’ perceptions of active teaching-learning methods and their effectiveness in enhancing engagement and academic outcomes. The questionnaire comprised six Likert-scale questions designed to evaluate various aspects of active learning, including engagement, retention, and critical thinking, along with three open-ended questions to capture qualitative feedback (Appendices). Thematic analysis was conducted manually through an iterative reading of the free-text responses. Keywords and recurring patterns were identified and grouped into themes using an inductive approach. Although qualitative analysis software can assist in coding, manual analysis was chosen due to the manageable volume of responses. The questionnaire was distributed using Google Forms on November 17, 2024. The form was configured to allow only one response per participant, ensuring data integrity by preventing multiple submissions. To ensure the reliability of the questionnaire, Cronbach’s alpha was calculated, yielding a value of 0.873, which reflects high internal consistency. A total of 147 questionnaires were distributed, of which 113 participants responded and were analyzed, yielding a response rate of 76.9%.

Data analysis

Quantitative data were analyzed using SPSS version 16 (SPSS Inc., Chicago, IL, USA), with descriptive statistics used to summarize findings. Open-ended responses were analyzed qualitatively to identify recurring patterns and suggestions.

## Results

The study included 113 participants, comprising 59 (52.21%) males and 54 (47.79%) females. The age distribution was as follows: 30.1% (n = 34) were between 17 and 19 years old, 62.83% (n = 71) were between 20 and 22 years old, 6.2% (n = 7) were between 23 and 25 years old, and 0.9% (n = 1) were in the age group of 26 years and above. Student perceptions of active teaching-learning strategies were assessed using six Likert-scale questions, with responses analyzed based on frequency and percentage distribution (Table 1).

**Table 1:** Frequency and percentage distribution of responses to Likert-scale questions regarding active learning.

Statement	Likert scale	n	Percentage (%)
1. Active teaching methods (group work, discussions, and interactive sessions) increase my engagement in class	Strongly Disagree	8	7.1
	Disagree	1	0.9
	Neutral	14	12.4
	Agree	61	54.0
	Strongly Agree	29	25.7
	Total	113	100.0
2. Self-directed learning promotes critical thinking	Strongly Disagree	7	6.2
	Disagree	4	3.5
	Neutral	40	35.4
	Agree	44	38.9
	Strongly Agree	18	15.9
	Total	113	100.0
3. Active learning methods help retain information better than traditional lectures	Strongly Disagree	4	3.5
	Neutral	24	21.2
	Agree	68	60.2
	Strongly Agree	17	15.0
	Total	113	100.0
4. Active learning techniques have improved my academic performance (grades, assessments)	Strongly Disagree	5	4.4
	Neutral	18	15.9
	Agree	78	69.0
	Strongly Agree	12	10.6
	Total	113	100.0
5. Active learning strategies are effective in developing clinical reasoning skills	Strongly Disagree	6	5.3
	Neutral	13	11.5
	Agree	80	70.8
	Strongly Agree	14	12.4
	Total	113	100.0
6. Incorporating more active learning strategies would improve performance in professional exams	Strongly Disagree	4	3.5
	Disagree	1	0.9
	Neutral	24	21.2
	Agree	69	61.1
	Strongly Agree	15	13.3
	Total	113	100.0

The study revealed several key observations regarding student perceptions of active teaching-learning strategies. A significant majority, 79.7% (n = 90), agreed or strongly agreed that active teaching methods enhanced their engagement in class, while only 8% (n = 9) expressed disagreement. Regarding critical thinking, 54.8% (n = 62) of students believed that self-directed learning fostered critical thinking skills, though 35.4% (n = 40) remained neutral. Active learning was also perceived as beneficial for information retention, with 75.2% (n = 85) of respondents endorsing its effectiveness, whereas only 3.5% (n = 4) strongly disagreed. Additionally, 79.6% (n = 90) of students reported that active learning techniques improved their academic performance. The role of active learning strategies in developing clinical reasoning skills was supported by 83.2% (n = 94) of participants, with 11.5% (n = 13) remaining neutral and 5.3% (n = 6) disagreeing. Moreover, 74.4% (n = 84) believed that integrating active learning strategies would enhance their performance in professional exams, while only 4.4% (n = 5) disagreed.

In the current study, qualitative insights were gathered through open-ended questions aimed at exploring perceived barriers to engagement in active teaching-learning methods and identifying suggestions to improve such engagement. Analysis of the open-ended responses revealed a strong preference for greater clinical exposure, often cited as “greater access to clinical simulations,” “exposure to clinical cases,” or “problem-based clinical case solutions.” This reflects a desire for contextual and application-oriented learning. Another major theme was the emphasis on small group discussions as a means to improve retention, engagement, and comfort in expression, with multiple students noting that “we can retain the concepts in a better way” and “we learn more easily from our friends.” A notable number of students also highlighted time constraints and an overcrowded curriculum, which they felt hindered deep learning; they recommended “active participation,” “one-to-one interaction,” and “peer-led sessions” to overcome these barriers. Several responses stressed the value of peer learning and student autonomy, with references to “self-directed learning,” “teacher-student co-interaction,” and “healthy debating.” Lastly, suggestions involving more flipped classrooms indicated an openness to blended and student-centered pedagogical innovations. These findings were categorized and quantified in Table 2 and Table 3.

**Table 2:** Barriers to effective student engagement in active learning methods. Of 113 participants, 93 provided valid responses to the open-ended question regarding barriers. As for the 20 non-responders, it was assumed that they did not identify any significant barriers, and their inputs were excluded from the thematic analysis.

Barrier	Count (n = 93)	Percentage (%)
Overcrowded curriculum	30	32.25
Time constraints	30	32.25
Lack of interest among students	13	14.00
Ineffective facilitation	13	14.00
Peer influence	7	7.52
Total	93	100.00

**Table 3:** Suggested methods to improve student engagement in active learning methods. Of the 113 respondents, four did not provide suggestions for improving engagement.

Improvement method	Count (n = 109)	Percentage (%)
Greater access to clinical simulations	57	52.30
More small group discussions	33	30.28
Increased feedback from instructors	9	8.26
Peer-led sessions	5	4.58
More flipped classroom sessions	5	4.58
Total	109	100.00

## Discussion

This study assessed the perceptions of first-year medical students regarding active teaching-learning strategies, with a focus on engagement, critical thinking, academic performance, and clinical reasoning. The findings suggest a strong overall endorsement of active learning methods among respondents. Active teaching-learning methods have gained momentum as transformative approaches in medical education. Student engagement is particularly critical in medical education, where passive content delivery may fail to stimulate deep learning or sustained interest.

Our study reinforces the notion that these methods significantly enhance student engagement, knowledge retention, and academic performance. The results align with a growing body of evidence supporting active learning in medical education. In our study, a majority of students, 79.7% (n = 90), agreed or strongly agreed that active teaching methods increased engagement. This finding mirrors a meta-analysis by Freeman et al., which reported that active learning strategies enhance student performance and reduce failure rates in STEM fields, including health sciences [5]. Patrick et al. reported that graduate students had limited exposure to active learning in their own coursework and believed that greater emphasis on such methods was warranted [6]. In contrast, teaching assistants generally felt they allocated sufficient time to active learning in the classes they instructed. Interestingly, graduate students acknowledged a discrepancy between the instructional approaches they employed while teaching undergraduates and those they preferred as learners, indicating a misalignment between teaching practices and personal learning preferences [6].

Additionally, our participants acknowledged the role of self-directed learning in promoting critical thinking (n = 62, 54.8%). This aligns with the findings of a mixed-methods systematic review by Berger-Estilita et al., highlighting the effectiveness of self-directed learning in fostering lifelong learning skills, a crucial competency for medical professionals [7]. Moreover, active methods such as problem-based learning and interactive small group discussions are reported to enhance students’ ability to synthesize information and apply it in clinical contexts [8,9]. In this study, 83.2% (n = 94) of the participants recognized active learning strategies as effective in developing clinical reasoning skills. Zhou et al., in a randomized controlled trial at Nantong University, found that while third-year medical students had comparable pre-test scores, those in the problem-based learning group showed significantly higher post-test scores and improved critical thinking subscales, with clinical thinking ability positively correlated with literature reading, self-directed problem-based learning learning, and problem-based learning performance [10].

Though the perception that self-directed learning promotes critical thinking was supported by more than half of the participants (n = 62, 54.8%), a substantial proportion (n = 40, 35.4%) of students remained neutral. This reflects a transitional phase in early medical education, where students are still adapting to the autonomy and responsibility required in self-directed learning. The moderate neutrality may indicate a need for better scaffolding or orientation in self-directed strategies to help students leverage them more effectively.

Retention of information was perceived as better with active learning approaches (75.2% agreement, n = 85), echoing cognitive theories that emphasize the effectiveness of active recall and application in strengthening memory and understanding. This result reinforces the argument for integrating active learning strategies such as case-based learning and problem-solving exercises into the curriculum. Studies have observed that active learning strategies, including retrieval practice, interleaved study, and self-explanation, have been consistently shown to enhance long-term retention and understanding of material. These methods engage students in deeper cognitive processing, facilitating better integration and recall of information [11,12]. For instance, retrieval practice not only reinforces memory but also aids in identifying knowledge gaps, allowing for targeted review and reinforcement. Interleaved study promotes discrimination between concepts, enhancing problem-solving skills, while self-explanation encourages learners to make connections between new information and existing knowledge frameworks [12].

In a study by Lawson et al., the effectiveness of active learning was evaluated by comparing exam performance between students who participated in optional microbiology lab sessions and those who did not. The lab activities, designed to reinforce key concepts through hands-on experience, offered students a learning opportunity that correlated with better outcome measures on the assessments. Over seven academic years, aggregated data showed that students who attended these sessions performed significantly better on exam questions directly related to lab content [13]. These findings highlight the value of active, collaborative learning environments in enhancing conceptual understanding and retention.

In this study, 79.6% (n = 90) of students believed that active learning methods improved their academic performance. This finding underlines the pedagogical value of active techniques in enhancing not only understanding but also assessment outcomes. Furthermore, 83.2% (n = 94) of students affirmed the role of active learning in developing clinical reasoning skills, an essential competency in medical education. This suggests that students recognize the transferability of active learning beyond the classroom into clinical practice and decision-making. McCoy et al. mapped active learning strategies to five core attributes of learner-centered education and demonstrated that such strategies promote engagement, clinical reasoning, collaboration, and self-assessment. Their study, conducted at a single institution, found that out of 646 hours of large group instruction in the first year, 476 (74%) hours incorporated at least one active learning component, such as discussion, case-based learning, debates, formative quizzes, audience response systems, and demonstrations [14].

Our study data suggest that active learning approaches have a direct impact on professional exam preparedness, as 74.4% (n = 84) of students expressed confidence in their ability to perform better due to these strategies. Lees-Murdock et al. implemented an active learning strategy by introducing weekly formative assessments using Smartwork-based interactive quizzes tailored to lecture content. Student performance was tracked for two years before and after this intervention to evaluate its impact across diverse academic backgrounds. Full engagement with the active learning approach significantly improved overall module performance and outcomes in class tests, coursework, and exams [15]. Lees-Murdock et al. further observed that partial engagement also led to notable gains in class test and coursework scores, although improvements in exam and overall marks did not reach statistical significance [15]. These findings suggest a clear positive association between active learning participation and academic performance. Such findings support curriculum designers’ efforts to integrate active learning techniques early in medical training. Incorporating these methods can improve not only academic outcomes but also essential competencies such as problem-solving, teamwork, and adaptability, which are critical in clinical practice.

The thematic analysis of open-ended responses provided valuable insights into the perceived barriers influencing student engagement in active learning methods among first-year medical students. The findings reveal critical themes that can guide educational reforms and promote learner-centered approaches in medical education.

A majority of students articulated clear barriers to effective participation in active teaching-learning methods. The most frequently reported obstacles were the overcrowded curriculum and time constraints, both of which reflect structural limitations inherent in the current undergraduate medical training framework. These issues suggest that students often feel overwhelmed by the volume and pace of academic content, leaving little room for reflection or active participation. Similar concerns have been echoed in previous literature, where rigid academic schedules and excessive factual burden have been shown to hinder learner engagement in constructivist methodologies [16].

In this study, a few participants (n = 13, 14%) implied that limited student engagement was associated with factors such as varying levels of interest and challenges in facilitation, highlighting the significance of both motivational and instructional elements. Ertmer et al. evaluated “problem space coverage” to compare learning outcomes in facilitated versus non-facilitated online case-based discussions, and their findings emphasized that good facilitation enhances the effectiveness of active learning for students [17].

In a study by Mohammed et al. in Ethiopia, implementation of problem-based learning as an active learning strategy was insufficient for improving students’ competence in basic science and clinical reasoning skills if the curriculum is poorly designed, the educators are inadequately trained, the resources are lacking, or the educational environment is unprepared [18]. This observation emphasizes that facilitator effectiveness and the ability to build rapport are closely linked to student engagement. In our study, a smaller subset of respondents cited peer influence as a barrier, indicating that social dynamics within groups can affect individual willingness to engage. This may reflect apprehensions about judgment, dominance by extroverted peers, or lack of collaborative spirit in group tasks.

With regard to improvements, the most popular suggestion, greater access to clinical simulations, reflects a desire for experiential, hands-on learning that mimics real-world medical practice. This finding reinforces the growing consensus that simulation-based learning can enhance clinical reasoning, decision-making, and engagement, especially among pre-clinical students [19]. The strong support for small group discussions further emphasizes the value of collaborative learning, which not only fosters deeper understanding but also encourages active participation among otherwise passive learners.

Other suggestions, such as increased feedback from instructors, peer-led sessions, and the flipped classroom model, point to students’ awareness of innovative pedagogical strategies and their preference for more interactive and feedback-rich learning environments. These insights are particularly encouraging, as they demonstrate a readiness among students to embrace progressive educational practices when adequately supported. Importantly, a small proportion of students (n = 20 for barriers and n = 4 for suggestions) did not respond to the open-ended questions.

Limitations and future directions

The study’s cross-sectional design and reliance on self-reported data are acknowledged limitations. Additionally, the sample size was limited to 113 first-year medical students from a single institution. While this number offers preliminary insights into student perceptions of active learning, it may not be fully representative of broader student populations. However, this focused sample was intentionally selected to ensure homogeneity in baseline exposure to teaching-learning methods and maintain feasibility for an in-depth exploratory study. Future research involving larger, multi-centric cohorts would enhance generalizability and support more nuanced comparisons across diverse educational contexts.

## Conclusions

By promoting critical thinking, improving information retention, and fostering clinical reasoning, active learning methods align with the competencies required of modern medical practitioners. The findings of this study reinforce the potential of active teaching-learning strategies in medical education, particularly in enhancing engagement, retention, and critical thinking. By addressing implementation challenges and fostering a culture of active learning, medical institutions can prepare students to meet the evolving demands of healthcare with confidence and competence. The integration of active learning into medical curricula should be prioritized, with emphasis on faculty training and resource allocation.

## Appendices

**Table 4:** Study questionnaire.

Section	Question number	Question	Options
Demographics	1	What is your age?	17–19 years, 20–22 years, 23–25 years, 26 years and above
	2	What is your gender?	Male, Female, Prefer not to say
Student Engagement	3	Active teaching methods (group work, discussions, interactive sessions) increase my engagement in class	Strongly agree, Agree, Neutral, Disagree, Strongly disagree
Learning Preferences	4	Self-directed learning promotes critical thinking	Strongly agree, Agree, Neutral, Disagree, Strongly disagree
Learning Outcomes	5	Active learning methods help retain information better than traditional lectures	Strongly agree, Agree, Neutral, Disagree, Strongly disagree
	6	Active learning techniques have improved my academic performance (grades, assessments)	Strongly agree, Agree, Neutral, Disagree, Strongly disagree
Educational Innovation and Adaptation	7	Active learning strategies are effective in developing clinical reasoning skills	Strongly agree, Agree, Neutral, Disagree, Strongly disagree
	8	Incorporating more active learning strategies would improve performance in professional exams	Strongly agree, Agree, Neutral, Disagree, Strongly disagree
Open-Ended	9	What barriers do you face in engaging with active learning techniques? (Select all that apply)	Open-ended
	10	What suggestions do you have to enhance student engagement in health education programs?	Open-ended
	11	How can the use of active teaching methods be improved in your current curriculum?	Open-ended
